# Gastrin stabilises *β*-catenin protein in mouse colorectal cancer cells

**DOI:** 10.1038/sj.bjc.6602509

**Published:** 2005-03-29

**Authors:** D H Song, J C Kaufman, L Borodyansky, C Albanese, R G Pestell, M Michael Wolfe

**Affiliations:** 1 Section of Gastroenterology, Boston University School of Medicine, Boston Medical Center, 650 Albany Street, Boston, MA 02118, USA; 2 Department of Oncology and the Lombardi Comprehensive Cancer Center, Georgetown University, Washington, DC 20057, USA

**Keywords:** MC-26 cells, G-17, *β*-catenin, cyclin D1

## Abstract

As gastrin may play a role in the pathophysiology of gastrointestinal (GI) malignancies, the elucidation of the mechanisms governing gastrin-induced proliferation has recently gained considerable interest. Several studies have reported that a large percentage of colorectal tumours overexpress or stabilise the *β*-catenin oncoprotein. We thus sought to determine whether gastrin might regulate *β*-catenin expression in colorectal tumour cells. Amidated gastrin-17 (G-17), one of the major circulating forms of gastrin, not only enhanced *β*-catenin protein expression, but also one of its target genes, cyclin D1. Furthermore, activation of *β*-catenin-dependent transcription by gastrin was confirmed by an increase in LEF-1 reporter activity, as well as enhanced cyclin D1 promoter activity. Finally, G-17 prolonged the *τ*_1/2_ of *β*-catenin protein, demonstrating that gastrin appears to exert its mitogenic effects on colorectal tumour cells, at least in part, by stabilising *β*-catenin.

Gastrin was originally described as a gastrointestinal (GI) regulatory peptide whose principal function was to stimulate postprandial gastric acid secretion. However, in addition to its recognised role in the physiological regulation of acid secretion, another biological property attributed to gastrin is its trophic effects. A prospective study by Thorburn *et al* (1998) suggested that hypergastrinemia is associated with an increased risk for colorectal cancer (CRC), and numerous studies have demonstrated that gastrin stimulates the growth of malignant colorectal adenocarcinomas (Wang *et al*, 1996; Malecka-Panas *et al*, 1997; Baldwin & Shulkes, 1998; Nakata *et al*, 1998; Koh *et al*, 1999; Stepan *et al*, 1999; Smith & Watson, 2000). Transgenic mice overexpressing progastrin and glycine-extended gastrin demonstrate enhanced colonic proliferation (Wang *et al*, 1996; Koh *et al*, 1999), and conversely, gastrin-deficient mice manifest decreased colonic proliferation (Koh *et al*, 1997). Repression of the gastrin gene in human colon cancer cells by antisense gastrin RNA yields a significant growth inhibition of these cells, suggesting that gastrin expression may be required for colon tumour progression (Singh *et al*, 1996). Although these studies all suggest a role for gastrin in the pathogenesis of CRC, little is known regarding the factors and mechanisms involved in mediating the trophic properties of this important peptide.

Overwhelming evidence derived from studies involving primary colon tumours of both hereditary and sporadic origin has implicated aberrations of the adenomatous polyposis coli (APC) tumour suppressor gene and *β*-catenin oncogene in the pathogenesis of CRC (Kinzler & Vogelstein, 1996; Mirabelli-Primdahl *et al*, 1999; Miyaki *et al*, 1999; Samowitz *et al*, 1999). Although multiple mechanisms may induce the neoplastic growth of colorectal tumours, *β*-catenin appears to play a pivotal role in this process. Under normal conditions, *β*-catenin degradation ensures tightly regulated cytoplasmic levels of this protein. Adenomatous polyposis coli appears to regulate the degradation of *β*-catenin protein by recruiting *β*-catenin into the negative regulatory complex for phosphorylation by glycogen synthase kinase 3*β* (GSK3*β*) in the N-terminus (Dominguez *et al*, 1995; Yost *et al*, 1996; Korinek *et al*, 1997) and subsequent proteasomal degradation (Aberle *et al*, 1997). Intricate interactions among other *β*-catenin binding partners also serve to facilitate the degradation of *β*-catenin to maintain a delicate balance (Ikeda *et al*, 1998; Kishida *et al*, 1998; Li *et al*, 1999; Farr *et al*, 2000). In contrast to the proteasomal degradation of *β*-catenin, which normally serves as a negative regulator of tumorigenesis, a positive regulator of *β*-catenin and tumorigenesis has also been identified. Protein kinase CK2 (formerly known as casein kinase 2), a serine/threonine kinase that is overexpressed in many malignancies, has been shown to phosphorylate *β*-catenin in the midportion of the protein and enhance its stability (Song *et al*, 2000, 2003a).

When *β*-catenin escapes its negative regulatory mechanisms, it translocates into the nucleus and functions as a critical transcriptional coactivator of the T-cell factor/lymphocyte enhancer binding factor (TCF/LEF), which activates oncogenes, such as c-*myc* (He *et al*, 1998) and cyclin D1 (Shtutman *et al*, 1999). Thus, activating mutations or stabilisation of *β*-catenin represent a critical process in the growth of the human CRC. Many *β*-catenin target genes have also been demonstrated as important factors in the pathogenesis of CRC. In particular, cyclin D1 was upregulated in human colorectal tumours and was associated with altered *β*-catenin expression (Wang *et al*, 2002). Moreover, increased levels of both *β*-catenin and cyclin D1 were found in a clinical analysis of tissue samples obtained from CRC patients (Utsunomiya *et al*, 2001) and in the colonic tissue extracts of mice when hyperproliferation/hyperplasia was induced (Sellin *et al*, 2001).

Interestingly, an association between gastrin and *β*-catenin was not made until Koh *et al* (2000) identified gastrin as a downstream target gene of *β*-catenin/TCF transcription. Because these factors are important contributors to CRC growth, we sought to determine whether additional relationships might exist between gastrin and *β*-catenin. We have previously demonstrated the trophic properties of gastrin in mouse colorectal tumour cells (MC-26), in which the peptide caused a significant incorporation of [^3^H]thymidine at 24 and 48 h (Yao *et al*, 2002). Furthermore, when MC-26 cells were injected subcutaneously into BALB/C mice and treated with amidated gastrin-17 (G-17) by continuous infusion, the weight and volume of resulting tumour tissues were significantly greater than in untreated controls (Yao *et al*, 2002).

In the present study, to determine whether the tropic properties of G-17 might involve modulation of *β*-catenin, MC-26 cells were treated with various concentrations of G-17, and various aspects of *β*-catenin expression were examined. We observed that G-17 not only enhanced the expression of total cellular *β*-catenin, but also increased nuclear *β*-catenin accumulation and *β*-catenin-dependent LEF-1 activity. Furthermore, cyclin D1 protein levels and promoter activity were enhanced by G-17. Finally, treatment with G-17 prolonged the half-life of *β*-catenin protein, suggesting that one of the major mechanisms by which G-17 might induce its trophic effects is through stabilisation of the multifunctional ongogenic *β*-catenin protein. The results of our studies are consistent with the presence of a vicious cycle between gastrin and *β*-catenin that would favour an environment for uncontrolled, aggressive CRC growth.

## MATERIALS AND METHODS

### Cell culture and treatments

We utilised MC-26 mouse CRC cells, which were maintained in Dulbecco's modified Eagle's Medium (DMEM; Gibco Laboratories, Grand Island, NY, USA), supplemented with 10% fetal bovine serum (Gibco) and 1% penicillin/streptomycin. Amidated G-17 (Peninsula/Bachem, Belmont, CA, USA) was added to the culture medium (20–100 nM) for 2–4 h, and 1 *μ*M of the gastrin-specific receptor antagonist L365,260 (kindly provided by Dr L Iverson, Oxford, UK) was used in conjunction with G-17 in the indicated experiments. Cycloheximide, a *de novo* protein synthesis inhibitor, was used at a final concentration of 10 *μ*g ml^−1^, either alone or in combination with 20 or 50 nM G-17 for the indicated times (0, 3, 6, and 24 h).

### Northern analysis

Total RNA was extracted using the Qiagen RNeasy kit (Qiagen Inc., Valencia, CA, USA) following the manufacturer's instructions. Each RNA sample (10 *μ*g) was loaded onto a formaldehyde-containing agarose gel and transferred via capillary action overnight onto a Hybond-N nylon membrane (Amersham Pharmacia, Piscataway, NJ, USA) in 10 × SSC buffer. Transferred membrane was crosslinked and prehybridised prior to the addition of the labelled probe. Approximately 1 kb fragment of *β*-catenin cDNA and 3.2 kb fragment of actin cDNA were excised and used as probes. Both of the probes were labelled with [^32^P]dCTP for 30 min, purified with Quick Spin sephadex G-25 columns (Roche, Basel, Switzerland), boiled, and incubated with prehybridised membrane overnight at 65°C. Labelled membranes were washed four times, twice in 2 × SSC/0.1% SDS and twice in 0.2 × SSC/0.1% SDS, before exposing to a film. The membrane was briefly stripped with boiling 0.1% SDS and washed 3 × with 2 × SSC before addition of another probe.

### Western analysis

Total protein was extracted, as previously described (Song *et al*, 2000, 2003a). For nuclear protein extraction, a protocol described by Dignam *et al* (1983) was followed. Briefly, cells were washed twice in 1 × phosphate-buffered saline (PBS) and scraped in the presence of 200 mM EDTA in PBS. Through Dounce homogenation and differential centrifugation, nuclear proteins were separated from cytoplasmic fractions in the presence of protease inhibitors. Protein quantification was performed using the BCA protein assay.

Western blotting analyses were performed, as previously described (Song *et al*, 2000, 2003a), using antibodies against *β*-catenin (Transduction Laboratories, Lexington, KY, USA) and cyclin D1 (Pharmingen, Chicago, IL, USA). Ponceau S (Sigma, St Louis, MO, USA) staining and immunoblots with either a monoclonal *β*-actin antibody (Sigma) for whole-cell lysates or polyclonal Sp1 antibody (Santa Cruz, Santa Cruz, CA, USA) for nuclear extracts were used to confirm equal loading of Western blot membranes.

### Reporter assays

MC-26 cells (1–2 × 10^5^) were plated 1 day prior to transfection. For transient transfection experiments, subconfluent cells were incubated with DNA and FUGENE-6 liposome reagent (Boehringer Mannheim, Mannheim, Germany) according to the manufacturer's instructions. Both LEF-1 and cyclin D1 (minimal and full-length cyclin D1 promoter-luciferase constructs; Albanese *et al*, 2003) experiments utilised the FUGENE-6 transfection reagent to deliver the target plasmids. In addition, renilla was cotransfected with the firefly reporter plasmids to normalise for transfection efficiency. Total DNA was balanced with the addition of empty vector when multiple plasmids were used. At 24 h after the transfection, the cells were incubated with 20 and 50 nM G-17 for 4 h, harvested, and assayed for firefly luciferase reporter and for renilla activity. Briefly, 10 *μ*l of protein extract was first assayed with 50 *μ*l of firefly luciferase substrate (Promega, Madison, WI, USA) in a luminometer for 10 s. Subsequently, in the same tube, 50 *μ*l renilla substrate (Promega) were added and measured. Samples were assayed in duplicate, and the luciferase counts were normalised to renilla measurements.

### *In vitro* kinase assay

Equal amounts of protein extracted from MC-26 cells (10 *μ*g) were assayed for CK2 kinase activity, as previously described (Song *et al*, 2000, 2003a). Briefly, each sample was assayed in duplicate with and without CK2-specific synthetic peptide, RRREEETEEE (Promega), for 20 min at 37°C with 5 *μ*Ci [*γ*-^32^P]ATP. Radioactive counts were blotted onto p81 filter circles, washed 4 × in 150 mM H_3_PO_4_, and analysed on an automated liquid scintillation counter. Buffer controls with and without the substrate peptide (background control) were also measured and subtracted from the final radioactive counts.

### Statistical analysis

The two-way Student's *t*-test was performed for paired comparisons. Statistical significance was assigned if *P*<0.05.

## RESULTS

### Gastrin-17 enhances *β*-catenin protein levels in MC-26 cells

**Figure 1:**
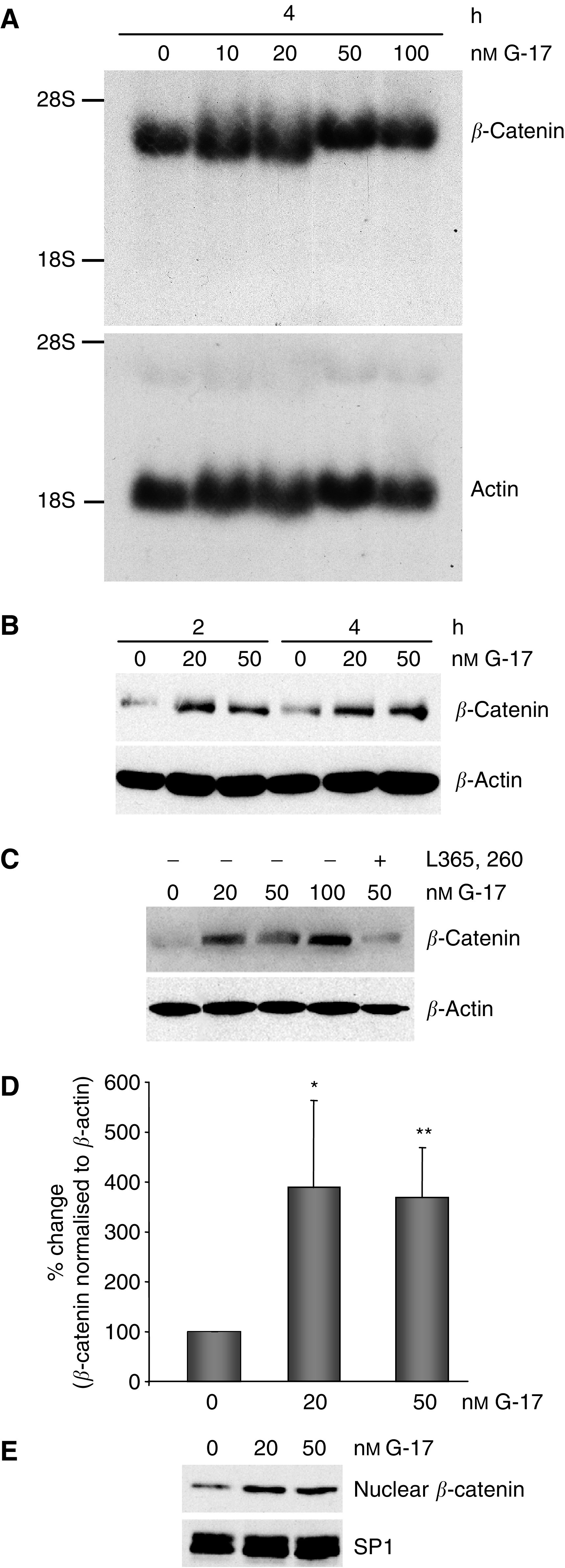
(**A**) Gastrin does not affect *β*-catenin mRNA levels, as demonstrated by Northern blot analysis. MC-26 cells were incubated for 4 h in the presence of increasing concentrations of G-17 (10–100 nM; upper panel). 28S and 18S ribosomal RNAs are indicated on the left, and actin was used as a loading control (lower panel). (**B**) Gastrin increases *β*-catenin protein levels, as demonstrated by Western blot analysis. Compared to untreated samples, 2 and 4 h of treatment with 20 and 50 nM G-17 caused a significant increase in *β*-catenin protein levels (upper panel). *β*-Actin was used as a loading control (lower panel). (**C**) The addition of 1 *μ*M L365,260, a gastrin-specific receptor antagonist, attenuated *β*-catenin induction by G-17 (upper panel, lane 5), indicating that the increase in *β*-catenin was gastrin-specific. (**D**) Average per cent change of *β*-catenin when normalised to *β*-actin levels, as measured by densitometry, of four independent experiments. Densitometry units were measured within each individual experiment and compared to control values, which were designated 100%, and per cent change in response to G-17 treatment was calculated. Data represent mean per cent of control±s.e. (*n*=4). ^*^P⩽0.05; ^**^P⩽0.01. (**E**) G-17 (20 and 50 nM) enhanced nuclear *β*-catenin levels (upper panel). Sp1, a nuclear protein, was used as a loading control (lower panel).

To determine the potential role of gastrin in modulating *β*-catenin, mRNA and protein levels were measured by Northern and Western blot hybridisations, respectively, in MC-26 cells that were transiently treated with G-17. Although incubation of MC-26 cells in the presence of G-17 did not alter the concentration of *β*-catenin transcripts (Figure 1A), total protein level of *β*-catenin was significantly enhanced by 20 and 50 nM G-17 following both 2 and 4 h of incubation (Figure 1B). Furthermore, coincubation with L365,260, a gastrin receptor (CCK2) antagonist, attenuated the upregulation of *β*-catenin by greater than 50%, suggesting that the increase in *β*-catenin was specific (Figure 1C). An induction of *β*-catenin was consistently detected, and the results were reproduced on four separate occasions. Although the magnitude of the change in total *β*-catenin protein varied within individual experiments, a 3–4-fold increase in total *β*-catenin protein levels was detected when bands were quantified by densitometry and normalised to *β*-actin levels (Figure 1D).

As mentioned above, nuclear accumulation of *β*-catenin represents a key event in CRC progression. To examine whether G-17 can enhance nuclear *β*-catenin in MC-26 cells, cells were treated with G-17 for 4 h and nuclear extracts were prepared. In all, 20 and 50 nM G-17 induced approximately a two-fold increase in nuclear *β*-catenin levels (Figure 1E), suggesting that G-17 promotes nuclear translocation of *β*-catenin. Expression of Sp1, a ubiquitously expressed transcription factor, was used as a loading control for nuclear extracts.

### Gastrin-17 increases LEF-1-dependent transcriptional activity

**Figure 2:**
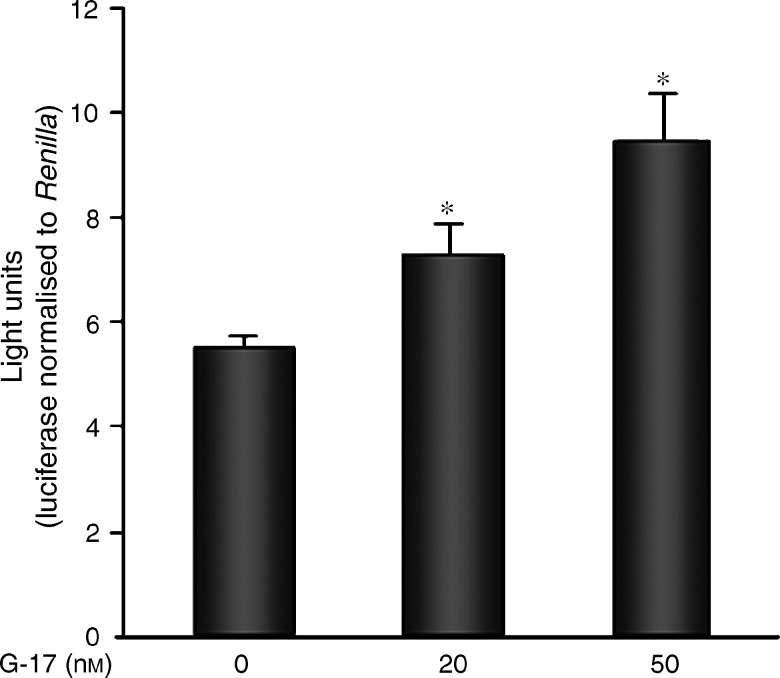
Gastrin-17 enhances LEF-1-dependent transcriptional activity. The representative figure shows LEF-1-dependent transcriptional activity (light units) normalised to renilla to control for transfection efficiency. Each sample was transfected and assayed in duplicate. ^*^P⩽0.005.

**Figure 3:**
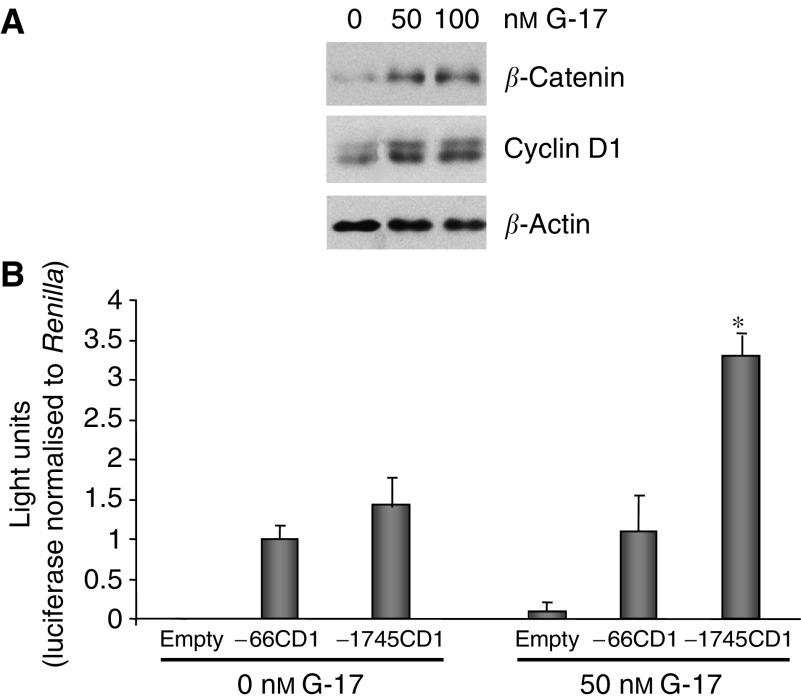
Gastrin-17 increases cyclin D1 protein and full-length promoter activity. (**A**) In addition to an increase in *β*-catenin levels, cyclin D1 protein levels were also enhanced by treatment with G-17 after 4 h. *β*-Actin was used as control for loading. (**B**) MC-26 cells transfected with empty, −66CD1 (minimal cyclin D1 promoter), or −1745CD1 (full-length cyclin D1 promoter) luciferase constructs for 24 h were either left untreated or treated with 50 nM G-17 for an additional 4 h. Renilla was cotransfected to control for transfection efficiency, and cells were examined for luciferase and renilla activity. While activity of the minimal promoter (−66CD1) was not altered, full-length promoter (−1745CD1) activity was markedly enhanced by 50 nM G-17. Each sample was transfected and assayed in duplicate. Values represent mean±s.e. (*n*=3). ^*^P⩽0.01.

To examine whether the increase in nuclear *β*-catenin protein is also associated with the activation of LEF-1, LEF-1-dependent reporter assays were performed. The pGL3-LEF-1 luciferase construct (kindly provided by Dr R Grosschedl, Munich, Germany) contains eight repeats of the LEF binding site that is activated only in the presence of an exogenous LEF-1 construct. The level of LEF-1-dependent transcription is also dependent on nuclear *β*-catenin levels, as *β*-catenin is a known coactivator for TCF/LEF transcription factors. As we speculated that an increase in *β*-catenin protein by G-17 might be functionally important for the transcriptional activation of its target genes, LEF-1-dependent reporter assays were performed in the absence and presence of G-17. We observed that 20 and 50 nM G-17 induced a concentration-dependent increase in LEF-1-dependent transcriptional activity (*P*⩽0.005) (Figure 2). In addition, the effects of gastrin on cyclin D1, one of the target genes of *β*-catenin-dependent transcription, were analysed. In response to the inclusion of G-17 in the culture medium, both cyclin D1 protein levels and promoter activity were increased (Figure 3). Specifically, 50 nM G-17 significantly enhanced the activity of the full-length cyclin D1 promoter (−1745) when compared to either the empty or minimal promoter (−66) (Figure 3B, *P*⩽0.01).

### Gastrin-17 stabilises *β*-catenin protein by increasing its half-life in MC-26 cells

**Figure 4:**
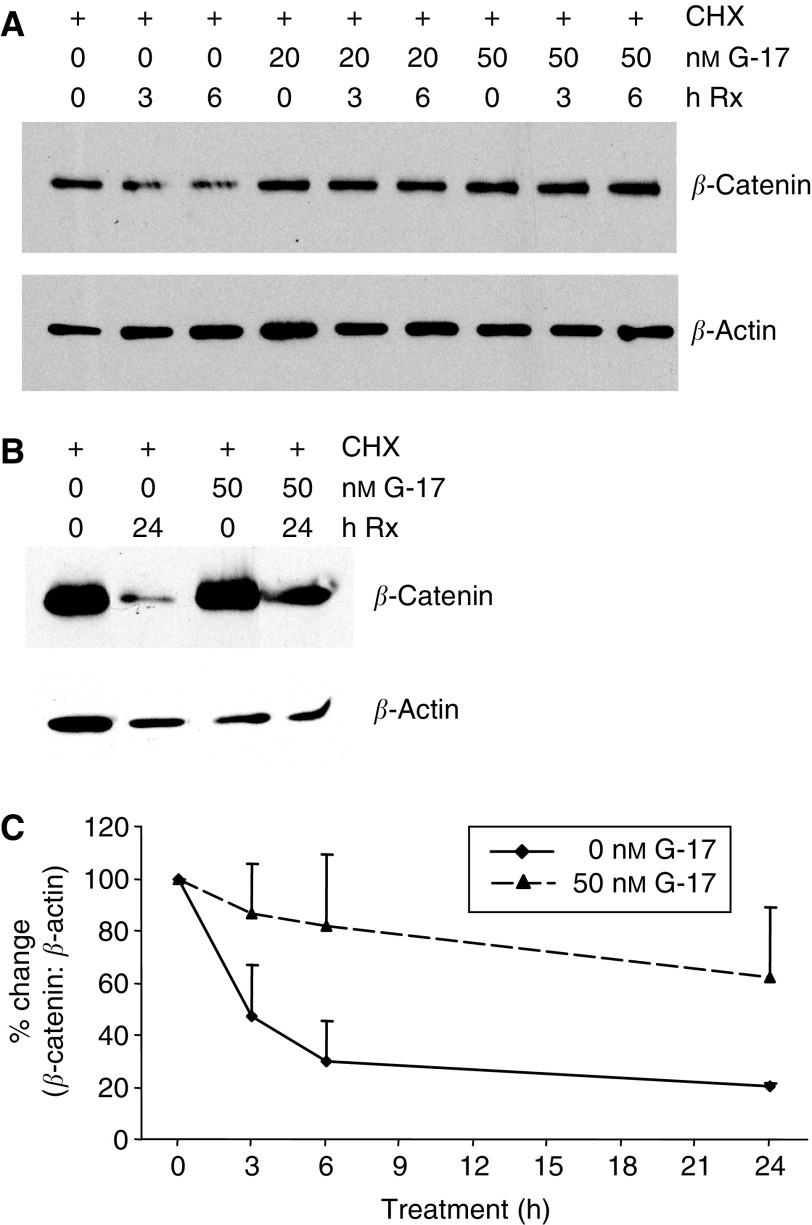
Gastrin-17 stabilises *β*-catenin. Equal amounts of MC-26 cells were treated with 10 *μ*g ml^−1^ cycloheximide (CHX) in the absence or presence of 20 or 50 nM G-17. (**A**) After 0, 3, and 6 h of treatment and (**B**) 0 and 24 h of treatment. In the presence of CHX only, *β*-catenin was degraded by approximately 50% after only 3 h. Coincubation of CHX with either 20 or 50 nM G-17 delayed the degradation of *β*-catenin. 50 nM G-17 caused a 50% reduction of *β*-catenin protein level after 24 h. *β*-Actin was used as a loading control. (**C**) Quantification of Western blot analysis examining *β*-catenin degradation. Values represent mean±s.e. (*n*=3). — Control; – – 50 nM G-17.

Since we observed that G-17 did not change the number of *β*-catenin transcripts but increased *β*-catenin protein levels, protein stability of *β*-catenin was next examined. To determine this, MC-26 cells were incubated with cycloheximide, a *de novo* protein synthesis inhibitor, either in the absence or presence of 20 and 50 nM G-17. Because *β*-catenin is normally degraded by proteasomes, the addition of cycloheximide would enable the pool of translated cytoplasmic protein to be degraded at its natural rate. Total protein was extracted at 0, 3, and 6 h, and *β*-catenin protein levels were measured by Western analysis. In the presence of cycloheximide alone, nearly 50% of *β*-catenin was degraded by 3 h (Figure 4A and C). However, coincubation with either 20 or 50 nM G-17 stabilised *β*-catenin and prolonged its half-life. Even after 6 h of treatment, *β*-catenin protein levels were largely unchanged in cells cultured in the presence of G-17 (Figure 4A). Specifically, the half-life of *β*-catenin in the presence of 50 nM G-17 was approximately 24 h, whereas nearly complete degradation of *β*-catenin protein was detected with cycloheximide alone at 24 h (Figure 4B and C). An approximate three-fold difference in *β*-catenin levels was detected between control conditions and following incubation in the presence of 50 nM G-17 at 24 h, suggesting that G-17 modulates *β*-catenin by stabilisation of the protein (Figure 4C).

**Figure 5:**
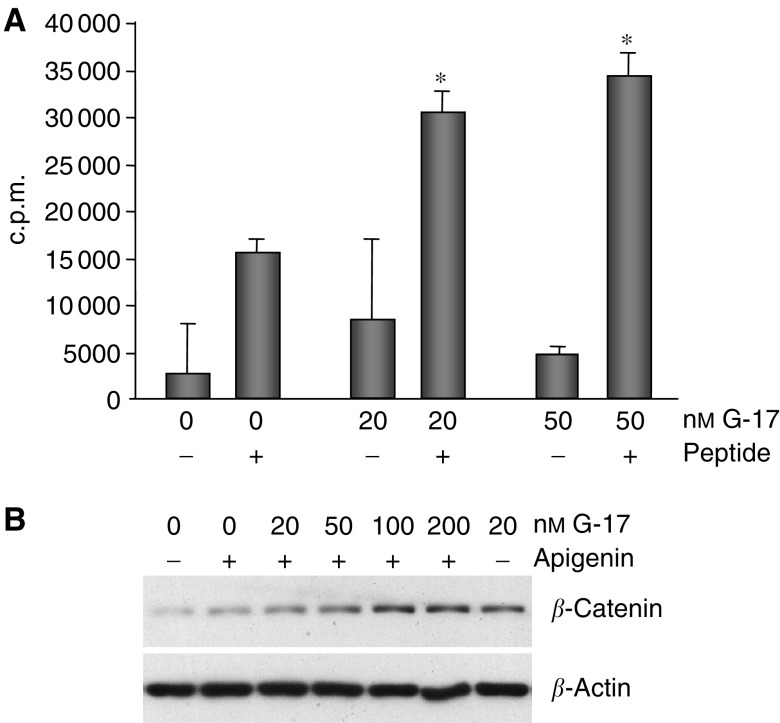
Gastrin-17 increases CK2 activity. (**A**) Endogenous protein kinase CK2 activity was examined by an *in vitro* kinase assay employing the measurement of [*γ*-^32^P]ATP incorporation into the CK2-specific substrate peptide RRREEETEEE (+peptide). In addition, in the same assay, background and nonpeptide-specific kinase activity was measured in the absence of the substrate (−peptide). Compared to untreated cells, 20 and 50 nM G-17 enhanced CK2 activity in the presence of the CK2 substrate. ^*^P⩽0.00003. (**B**) MC-26 cells were either treated for 5 h with 80 *μ*M apigenin alone or pretreated for 1 h with 80 *μ*M apigenin followed by 4 h of co-incubation with various concentrations of G-17. Total protein levels of *β*-catenin as well as *β*-actin loading control are shown. Treatment with 20 nM G-17 alone (lane 7) caused more *β*-catenin accumulation than coincubation of 20 nM G-17 and apigenin (lane 3). However, the addition of apigenin did not completely inhibit the effects of G-17 on *β*-catenin (lanes 2–6), suggesting that G-17 utilises multiple pathways in modulating this important protein, including its positive regulator, protein kinase CK2.

To delineate the mechanism by which gastrin might cause stabilisation of *β*-catenin, we examined two known regulators of *β*-catenin. Specifically, GSK3*β*, an upstream negative regulator of *β*-catenin that promotes proteasomal degradation of *β*-catenin, and protein kinase CK2, a positive regulator, were examined. No consistent effect on GSK3*β* kinase activity could be demonstrated in response to the incubation of MC-26 cells in media containing various concentrations of G-17 (data not shown). In contrast, 20 and 50 nM G-17 caused a marked increase in endogenous CK2 kinase activity (Figure 5A). Moreover, coincubation of 20 nM G-17 with apigenin, a purportedly selective CK2 inhibitor, attenuated total *β*-catenin protein levels compared to 20 nM G-17 alone, suggesting that G-17 may utilise CK2 to regulate *β*-catenin (Figure 5B). However, the addition of apigenin to media containing increasing concentrations of G-17 did not abolish the induction of *β*-catenin (Figure 5B).

## DISCUSSION

Both gastrin and various components of the *β*-catenin-dependent signaling pathway have been implicated in the pathogenesis of CRC (Nusse, 2002). However, a functionally relevant association between gastrin and *β*-catenin was not made until Koh *et al* (1997, 2000) demonstrated that gastrin-deficient APC (min^−/+^) mice produced fewer polyps than APC (min^−/+^) mice overexpressing gastrin. Furthermore, these investigators showed that *β*-catenin enhanced gastrin promoter activity, thus identifying gastrin as one of its numerous downstream targets. However, the possibility of a positive feedback relationship between gastrin and *β*-catenin expression has not been examined previously.

Utilising transplantable mouse CRC cells (MC-26) that express functional gastrin receptors, we have previously demonstrated the trophic properties of gastrin (Yao *et al*, 2002). We hypothesised that one of the mechanisms by which gastrin might exert its trophic properties may involve the multifunctional *β*-catenin protein. We consistently observed that gastrin increases *β*-catenin protein levels. However, despite our attempts to maintain consistency, such as plating equal amounts of cells 1 day prior to each individual experiment, we nevertheless did observe some variability in the basal expression (untreated) of *β*-catenin during the performance of different experiments. This variability may be due in part to the role of *β*-catenin in cell–cell adhesion, which, depending on cell density, may contribute to the variability in basal *β*-catenin expression.

Further examination of MC-26 cells in the present study suggests that gastrin prolongs the half-life of *β*-catenin by increasing its stability. Thus, it appears that *β*-catenin enhances gastrin expression, and conversely, *β*-catenin protein expression is stabilised by gastrin, completing a vicious cycle that may contribute to neoplastic cell survival and growth. Moreover, data presented in this study provide further evidence for the complex nature of the oncogenic process by suggesting that gastrin utilises multiple pathways in regulating *β*-catenin. In the present study, we observed that gastrin stimulated CK2 activity and that gastrin-stimulated *β*-catenin expression was partially attenuated in the presence of the CK2 selective inhibitor apigenin. Inhibition of CK2 activity did not abolish gastrin-mediated effects on *β*-catenin, suggesting that gastrin signalling possesses both CK2-dependent and -independent properties. It is plausible that this important regulatory peptide controls *β*-catenin through multiple regulators, as *β*-catenin itself is known to have numerous modulators (Dominguez *et al*, 1995; Yost *et al*, 1996; Korinek *et al*, 1997; Ikeda *et al*, 1998; Kishida *et al*, 1998; Li *et al*, 1999; Farr *et al*, 2000; Song *et al*, 2000, 2003a).

The existence of a pathological vicious cycle (a positive feedback loop) involving *β*-catenin, as we postulate, would serve to enhance the survival and continued growth of CRC cells by selectively upregulating various oncogenic factors. A recent study has suggested the existence of another pathological vicious cycle involving *β*-catenin in CRC. In addition to its effects on gastrin and on the expression of other target genes, Hovanes *et al* (2001) reported that LEF-1, one of the transcriptional partners of *β*-catenin, is likewise a target gene of *β*-catenin/TCF-dependent transcription.

The upregulation of *β*-catenin expression by gastrin was also associated with the enhancement of the critical cell cycle regulator, cyclin D1. Consistent with our current findings, we have previously reported that gastrin enhanced cyclin D1 protein and cyclin D1 promoter activity in the human gastric adenocarcinoma cell line AGS-B (Song *et al*, 2003b). However, in contrast to the present study, we did not observe an increase in *β*-catenin protein expression in AGS-B cells incubated in the presence of gastrin. Several possibilities may explain these disparate results, including interspecies variations. Another possibility is the fact that AGS-B cells have been engineered to overexpress the gastrin receptor, which could potentially favour a direct increase in cyclin D1 by gastrin rather than utilising *β*-catenin as a mediator of transcription. Furthermore, overexpression of the receptor may have modulated other components that could affect *β*-catenin stability. Despite our observation in the present study that both *β*-catenin and cyclin D1 expression were enhanced by gastrin, it is nevertheless possible that the increase in cyclin D1 may have occurred independently of *β*-catenin-dependent transcription. Along these lines, gastrin has been previously shown to stimulate the expression of c-*myc*, another target of *β*-catenin, in intestinal epithelial cells (IEC-6) (Wang *et al*, 1995). Although we did not examine c-*myc* levels in this study, it is certainly possible that gastrin may involve not only c-*myc* and cyclin D1, but also multiple *β*-catenin target genes and pathways in exerting its growth potential, whether directly or indirectly. Another possibility is simply the fact that every immortal cell line possesses slightly different characteristics that produce disparate results. For example, unlike AGS-B cells (Song *et al*, 2003b), in AGS-E cells, a related human gastric adenocarcinoma cell line overexpressing the gastrin receptor, G-17 induction of cyclin D1 transcription was mediated through both *β*-catenin and CREB pathways (Pradeep *et al*, 2004).

In conclusion, the results of the present studies demonstrate for the first time that gastrin enhances *β*-catenin protein by prolonging its half-life. Furthermore, these studies support our hypothesis that a positive feedback mechanism exists between gastrin and *β*-catenin. Although further studies will be required to elucidate fully the mechanisms governing gastrin-induced cellular proliferation, our results suggest that through aberrant overexpression of *β*-catenin, malignant cells appear to amplify various signals via positive feedback between molecules as a means for potentiating tumorigenesis and proliferation.
